# Lymph Node Lymphatic PD-L1 Regulates Tumor-Infiltrating T Cells in Immune Checkpoint–Sensitive Melanoma

**DOI:** 10.1158/2767-9764.CRC-26-0666

**Published:** 2026-10-23

**Authors:** Emma Reynaud, Sonya Belherazem, Shruthi Hemanna, Sarah Hofmann, Alperen Acari, Pragati Lodha, Özge Cicek Sener, Ziyuan Wang, Sebastian A. Wohlfeil, Cyrill Géraud, Jochen Utikal, Margaret Tulessin, Michael Detmar, Carolin Mogler, Lothar C. Dieterich

**Affiliations:** 1 European Center for Angioscience (ECAS), Medical Faculty Mannheim, Heidelberg University, Heidelberg, Germany.; 2 Mannheim Institute of Innate Immunoscience (MI3), Medical Faculty Mannheim, Heidelberg University, Heidelberg, Germany.; 3 Heidelberg Bioscience International Graduate School (HBIGS), Faculty of Biosciences, Heidelberg University, Heidelberg, Germany.; 4 Department of Dermatology, Venereology and Allergology, University Medicinie Mannheim, Mannheim, Germany.; 5 DKFZ Hector Cancer Institute, University Medical Center Mannheim, Mannheim, Germany.; 6 Skin Cancer Unit, German Cancer Research Center (DKFZ), Heidelberg, Germany.; 7 Section of Clinical and Molecular Dermatology, Medical Faculty Mannheim, Heidelberg University, Mannheim, Germany.; 8 Institute of Pathology, School of Medicine and Health, Technical University of Munich (TUM), Munich, Germany.; 9 Institute of Pharmaceutical Sciences, Swiss Federal Institute of Technology (ETH) Zurich, Zurich, Switzerland.

## Abstract

Lymph node (LN)–resident lymphatic endothelial cells (LEC) form the interphase between the lymphatic sinus lumen and the LN cortex. Beyond their role in leukocyte trafficking, LECs shape immune responses as they scavenge and cross-present exogenous antigens from the afferent lymph and constitutively express immune-regulatory proteins such as PD-L1. However, it remains unclear which immune cell types and activation states are sensitive to LN lymphatic PD-L1, and whether lymphatic PD-L1 expression is relevant for immune checkpoint blockade (ICB) sensitivity in patients with cancer. To dissect the contribution of LN lymphatic PD-L1 to tumor immunity and ICB outcomes in melanoma, we utilized a conditional lymphatic PD-L1–knockout mouse model in combination with syngeneic melanoma models varying in ICB sensitivity. Although lymphatic PD-L1 deletion did not alter primary tumor growth, progression, or the overall immune landscape within tumor-draining LNs, it modulated the intratumoral immune composition in ICB-sensitive but not in ICB-resistant melanoma. Specifically, lymphatic PD-L1 deletion resulted in an increased frequency of proliferating PD-1^+^ CD8^+^ T cells and a reduction in PD-1^+^ Th1 T cells. Together, our findings reveal that lymphatic PD-L1 plays a context-dependent role in shaping antitumor T cell responses, particularly in ICB-sensitive disease.

**Significance:**

The authors expand the concept of immune checkpoint regulation beyond immune cells in the tumor microenvironment, demonstrating how lymphatic endothelial PD-L1 in melanoma-draining LNs regulates tumor-infiltrating T cells in immune checkpoint–sensitive melanoma.

## Introduction

Immune checkpoint blockade (ICB) was initially conceived to reinvigorate exhausted T cells in the tumor microenvironment (TME) and restore antitumor effector functions (1). However, accumulating evidence indicates that ICB exerts effects not only within the TME but also regionally and systemically (2, 3). In particular, tumor-draining lymph nodes (tdLN) have emerged as critical sites of action in ICB therapy. Notably, lymphadenectomy performed in mice prior to ICB treatment abolishes its therapeutic efficacy, underscoring the importance of tdLNs in mediating treatment responses (3, 4). Moreover, localized administration of ICB to tdLNs yields equal or superior tumor control compared with systemic treatment (5–7). Several studies have identified early-stage CD8^+^ T cells within tdLNs as direct responders (R) to ICB. By expressing TCF1 and maintaining strong proliferative and self-renewal capacities, these cells generate a robust pool of TCF1^+^ TOX^+^ progenitor exhausted T cells (T_pex_) that subsequently migrate to the TME (3, 8).

LNs consist of diverse immune and stromal cell populations, including lymphatic endothelial cells (LEC). Interestingly, LECs constitutively express immune checkpoint proteins such as PD-L1 (CD274), while lacking costimulatory molecules, suggesting a potential role in peripheral tolerance and immune suppression (9–14). PD-L1 mRNA is robustly expressed by lymph node (LN) LECs, specifically within subcapsular sinus floor (fLEC) and medullary sinus (mLEC) subsets, in contrast to peripheral LECs in which PD-L1 is absent in steady state and is only upregulated in response to inflammation (15–18). Because of their location, LN LECs are in direct contact with migrating and resident immune cells, including macrophages, dendritic cells, and lymphocytes, potentially influencing a broad spectrum of immune responses.

We previously demonstrated that lymphatic PD-L1 restrains tumor antigen-specific CD8^+^ T cells with a CD44^+^ CD62L^+^ central memory phenotype in mouse models of ICB-sensitive colorectal carcinoma (MC38) and ICB-resistant melanoma (B16-F10), indicating that LN LECs contribute to tumor immune evasion via expression of PD-L1 (19). However, the precise impact of lymphatic PD-L1 on the immune landscape of tdLNs and the TME, and its relation to tumor ICB responsiveness, is not well understood. Furthermore, although LN LECs express PD-L1 mRNA in both mice and humans, the presence of PD-L1 protein in human tdLN LECs has not been investigated.

To address these questions, we used multiplex immunostaining to confirm that PD-L1 is expressed by fLECs and mLECs in human melanoma–draining LNs, which correlates with ICB responsiveness, whereas subcapsular sinus (SCS) ceiling LECs (cLEC) exhibited minimal to no PD-L1 expression. Furthermore, we comprehensively charted the immunologic changes following lymphatic PD-L1 deletion within the tdLNs and primary tumors of ICB-sensitive and -resistant melanoma models in mice using multidimensional flow cytometry analysis. Although the deletion of lymphatic PD-L1 did not affect tumor growth or progression, we observed changes in the T cell infiltrate exclusively in the TME of ICB-sensitive tumors, including expansion of proliferating PD-1^+^ CD8^+^ T cells and reduction of PD-1^hi^ Th1 cells, suggestive of an improved antitumor T cell response. Thus, this study provides new insights into how lymphatic PD-L1 influences antitumor immunity and how it may affect the outcomes of ICB therapy in melanoma.

## Materials and Methods

### Mice

All animal experiments in this study were approved by the responsible regional authorities (Regierungspräsidium Karlsruhe, permit G-177/22). PD-L1^LECKO^ mice, generated by crossing PD-L1^flox^ mice (20) backcrossed to a C57BL/6N background with Prox1-CreER^T2^ mice (21), have been described previously (19). Male mice were used to match the sex of the tumor cell lines. Cre-mediated recombination was initiated by treating 7-week-old mice with tamoxifen (50 mg/kg) in sunflower oil for five consecutive days intraperitoneally. Cre- littermate mice served as controls and were equally injected with tamoxifen. The mice had free access to food and drinking water.

### Tumor models

Three days after the last injection, mice were intradermally injected with tumor cells under isoflurane anesthesia. The following cell numbers were injected intradermally in both flanks: 2 × 10^5^ YUMM1.7 and YUMM5.2 cells and 2 × 10^6^ YUMMER1.7 cells, in a volume of 25 μL of PBS. Mice were monitored three times per week, with tumor growth assessed through caliper measurements. Mice were euthanized by cervical dislocation when tumors reached a volume of 1 cm^3^.

Inhibition of T cell egress from tdLNs was achieved by intraperitoneal injection of FTY720 (1 mg/kg, Sigma-Aldrich, cat. #SML0700) or vehicle control (sterile water). The FTY720 treatment started on day 7 after inoculation of tumor cells and continued for a total of three injections. Mice were euthanized on day 10 after inoculation and tissues were harvested for analysis.

### Cell lines

YUMMER1.7 cells (RRID: CVCL_A2AX; ref. 22) were kindly provided by Prof. Cornelia Halin (Swiss Federal Institute of Technology Zurich, Zurich, Switzerland) in 2023, and the YUMM1.7 and YUMM5.2 cell lines (RRID: CVCL_JK16 and CVCL_JK43; ref. 23) were generously provided by Prof. Jonathan Sleeman (Medical Faculty Mannheim, Heidelberg University, Mannheim, Germany) in 2024. All cell lines were cultured in DMEM/F12 media supplemented with 1% nonessential amino acids and 10% FBS. Cells were thawed from a fresh vial 1 week prior to each set of experiments and expanded for a maximum of five passages. Cell lines were routinely tested for *Mycoplasma* contamination using the Mycoplasma PCR Detection kit (Abcam, cat. #AB289834-1002), and all cells used in this study were tested negative. The cell lines were not authenticated as reference short tandem repeat profiles were partially unavailable.

### Tissue processing

Tumor-draining inguinal and axillary LNs, along with primary tumors from both flanks of the mice, were collected. One inguinal LN was cryopreserved for immunofluorescence analysis whereas the remaining LNs were prepared for flow cytometry analysis. To process the LNs, the capsule was disrupted using a needle followed by enzymatic digestion in a solution containing 1 mg/mL Collagenase type IV (Thermo Fisher Scientific) and 40 μg/mL DNase I (Roche) in DMEM supplemented with 2% FBS and 1.2 mmol/L CaCl_2_ at 37°C for 20 minutes. After this first digestion, the supernatant was collected, and the residual LN fragments underwent a second digestion using 3.5 mg/mL Collagenase type IV and 40 μg/mL DNase I in DMEM supplemented with 2% FBS and 1.2 mmol/L CaCl_2_ at 37°C for 15 minutes. The resulting cell suspensions were passed through a cell strainer and resuspended in FACS buffer (1% FBS, 1 mmol/L EDTA, and 0.02% NaN_3_ in PBS) with anti-CD16/CD32 (BioLegend, cat. #101302, RRID: AB_312801) and kept on ice.

For tumor processing, the tumor tissue was minced and enzymatically digested with 3.5 mg/mL Collagenase type IV and 40 μg/mL DNase I in DMEM supplemented with 2% FBS and 1.2 mmol/L CaCl_2_ at 37°C for 30 minutes. The digested tumors were filtered and passed through a 40-μm cell strainer, and the cell suspension was subjected to an erythrocyte lysis buffer (0.15 mol/L NH_4_Cl, 0.01 mol/L KHCO_3_, and 0.1 mmol/L Na_2_EDTA). Finally, cells were resuspended in FACS buffer with anti-CD16/CD32 and kept on ice.

### Flow cytometry

Cell suspensions were stained with surface antibodies (see Supplementary Table S1) in 50 μL of Brilliant Stain Buffer (BD Biosciences) for an hour on ice. Following surface staining, cells were fixed and permeabilized overnight using the Foxp3 Transcription Factor Staining Buffer Set (Thermo Fisher Scientific) for intracellular staining (see Supplementary Table S1). Data acquisition was performed using a NothernLight instrument (Cytek, RRID: SCR_027072) and data analysis using FlowJo (v10.10.0, BD Biosciences, RRID: SCR_008520).

High-parametric flow cytometry data analysis, including projection and clustering, was conducted as described by Ingelfinger and colleagues (24). Unmixed flow cytometry data were compensated in FlowJo, and immune cell populations (CD3^+^CD8^+^, CD3^+^CD4^+^, CD3^−^CD19^+^, and CD11b^+^ and/or CD11c^+^) were gated manually. Whenever possible, 10,000 cells per sample were exported. If fewer were available, all cells were included. Exported cell populations were analyzed in RStudio (v4.4.2, RRID: SCR_000432). The CyCombine package was used to merge data from the different mouse model experiments. Prior to dimensionality reduction, data were arcsinh-transformed and normalized to a 0 to 1 range. Uniform Manifold Approximation and Projection (UMAP) was applied for visualization, and unsupervised clustering was performed using the FlowSOM algorithm (RRID: SCR_016899). The number of clusters was determined based on the marker projection within the UMAP to ensure relevant clustering. Data visualization was carried our using the ggplot2 package (RRID: SCR_014601) and statistical tests using Student *t* test analysis.

### Immunofluorescence staining and analysis of tdLNs (mouse)

LNs were fixed in 4% paraformaldehyde for 2 hours, washed once in PBS, and incubated overnight in 30% sucrose before being embedded in optimal cutting temperature compound (Tissue-Tek) and cryopreserved at −80°C. Tissue sections of 7 μm were fixed using acetone and 80% methanol following rehydration in PBS. For staining, sections were incubated for 1 hour in a blocking solution containing 5% donkey serum, 1% BSA, 0.05% NaN_3_, and 0.1% Triton X-100 in PBS. Primary antibodies were then applied for an hour in the same blocking solution. The primary antibodies used were rabbit anti-*BRAF*^V600E^ (Thermo Fisher Scientific, cat. #MA5-24661, RRID: AB_2661889) and anti–CD45–biotin (BioLegend, cat. #103104, RRID: AB_312969). After washing, sections were incubated for an hour with the secondary antibodies: donkey anti–rabbit AF594 (Thermo Fisher Scientific, cat. #A-21207, RRID: AB_141637), streptavidin-AF488 (BioLegend, cat. #405235), and Hoechst 33342 (Thermo Fisher Scientific, cat. #H3569, RRID: AB_2651133) in PBS. Sections were then washed, mounted, and imaged using an Axioscan 7 (Zeiss) scanner. To quantify BRAF^V600E^^+^ metastatic areas in YUMM5.2-, YUMM1.7-, and YUMMER1.7-draining LNs, multiple LN sections spaced approximately 100 μm apart were stained for CD45 and BRAF^V600E^. Naïve LNs were used as control. Merged immunofluorescence channels were used to identify CD45^−^/BRAF^V600E^^+^ cell clusters. From the metastatic regions manually outlined, a particle analysis was performed, and the results were normalized to the dimensions of a predefined region of interest (ROI) covering the whole LN cross-section.

### Multiplex imaging of formalin-fixed, paraffin-embedded human melanoma–draining LNs

The use of human samples in this study was approved by the Technical University of Munich (TUM) Ethics Committee (approval number: 2022-140-1-S-KH), and approvals 2010-318N-MA and 2014-835R-MA by the ethics committee II of Heidelberg University, Germany. Written informed consent was obtained from each individual donating tissue according to the Declaration of Helsinki. Formalin-fixed, paraffin-embedded (FFPE) sections of locoregional metastatic, melanoma-draining LNs collected from three patients at the Department of Pathology, TUM, were dewaxed and rehydrated, following epitope retrieval with EDTA or citrate buffer, respectively. Multiplex fluorescence staining was performed using the PhenoCycler instrument (Akoya Biosciences, RRID: SCR_023773) using rabbit anti-PROX1 (Abcam, cat. #ab199359, RRID: AB_2868427), mouse anti-podoplanin (Agilent, cat. #M3619, RRID: AB_2162081), rabbit anti-PRAME (Cell Signaling Technology, cat. #56426, RRID: AB_2799510), rabbit anti-NT5E (Cell Signaling Technology, cat. #13160, RRID: AB_2716625), and rabbit anti–PD-L1 (Cell Signaling Technology, cat. #13684, RRID: AB_2687655), followed by corresponding OPAL detection reagents.

Images were analyzed using QuPath (RRID: SCR_018257). ROIs including the total LN area, excluding metastasis regions, and the SCS area were selected. Then, LECs were identified by detecting PROX1^+^ nuclei using a defined fluorescence intensity threshold specific to that LN section. A 2-μm perimeter was then expanded around each detected nucleus to include the surrounding cytoplasmic area. Nucleus detection parameters were set to a minimum area of 1 μm^2^, a maximum area of 1,000 μm^2^, and a sigma value of 2 μm. For each PROX1^+^ event, the mean fluorescence intensity of all relevant channels was exported. In R, exported values were classified into LEC subsets based on CD73 and podoplanin (D2-40) intensity, with thresholds for each marker defined separately for every LN section. PD-L1 mean expression values were normalized to the double-negative (CD73^−^ podoplanin^–^) subset and then analyzed across subsets using GraphPad Prism.

To quantify lymphatic PD-L1 in melanoma-draining LNs of responder (R) and nonresponder (NR) patients, we stained FFPE sections of melanoma-draining LNs collected from 10 patients [*n* = 6 (NR) vs. *n* = 4 (R)] who received nivolumab (anti–PD-1) and in some cases also ipilimumab (anti-CTLA4) in an adjuvant setting (Supplementary Table S2). The slides were baked for 30 minutes at 60°C before a deparaffinization step with a succession of xylene and ethanol baths. The slides were then incubated in a Tris-EDTA (pH 9) epitope retrieval buffer at 97°C for 10 minutes and were allowed to cool down for 20 minutes in the buffer before the blocking step. The slides were then stained for claudin-5 (Thermo Fisher Scientific, cat. #35-2500, RRID: AB_2533200) and PD-L1 (Cell Signaling Technology, cat. #13684, RRID: AB_2687655), followed by corresponding secondary antibodies [donkey anti–mouse AF594 (Thermo Fisher Scientific, cat. #A-11005, RRID: AB_2534073), donkey anti–rabbit AF647 (Thermo Fisher Scientific, cat. #A-31573, RRID: AB_2536183)], and Hoechst 33342 (Thermo Fisher Scientific, cat. #H3569, RRID: AB_2651133). Finally, the sections were mounted and scanned using a STELLARIS 8 confocal microscope (Leica Microsystems, RRID: SCR_024664). The images were analyzed using Fiji (RRID: SCR_002285; ref. 25). To quantify PD-L1 on lymphatic vessels, an ROI encircling the total LN area and an ROI below the SCS area were drawn. Before quantification, the outside region, damaged areas, and staining artefacts were cleared from the image. The image was then split into separate fluorescence channels. After adjusting and applying an appropriate threshold for the claudin-5 channel, a binary image was created and processed using the erode and dilate functions to create a mask of merged vascular structures. Lastly, structures >50 μm^2^ were selected and projected onto the PD-L1 channel to measure their mean PD-L1 staining intensity. The SCS area and medullary/cortical regions were analyzed separately using this approach. Areas with visibly low to absent PD-L1 staining intensity were used to determine the background signal.

### The Cancer Genome Atlas data analysis

Correlation between LN *CD274* expression and overall survival of patients with melanoma [Skin cutaneous melanoma (SKCM) cohort] was investigated using the UCSC Xena browser (RRID: SCR_018938; ref. 26). The median expression level was used as cutoff to stratify into low- and high-expressing patients. Statistical analysis was done using a log-rank test.

### Statistics

GraphPad Prism (v10.2.3, RRID: SCR_002798) and RStudio (v4.4.2, RRID: SCR_000432) were utilized for statistical tests. Statistical significance is indicated in the figures by asterisks; where no significance annotation is shown, the comparison was not statistically significant. The statistical test performed for each experiment and results are included in the figure legends. A *P* value of less than 0.05 was considered statistically significant. One Cre+ mouse was excluded from the FTY720 study after being identified as an outlier in terms of tumor T-cell infiltration using the ROUT method (Q = 1%).

## Results

### PD-L1 is expressed by fLECs and mLECs in human melanoma–draining LNs

**Figure 1.**
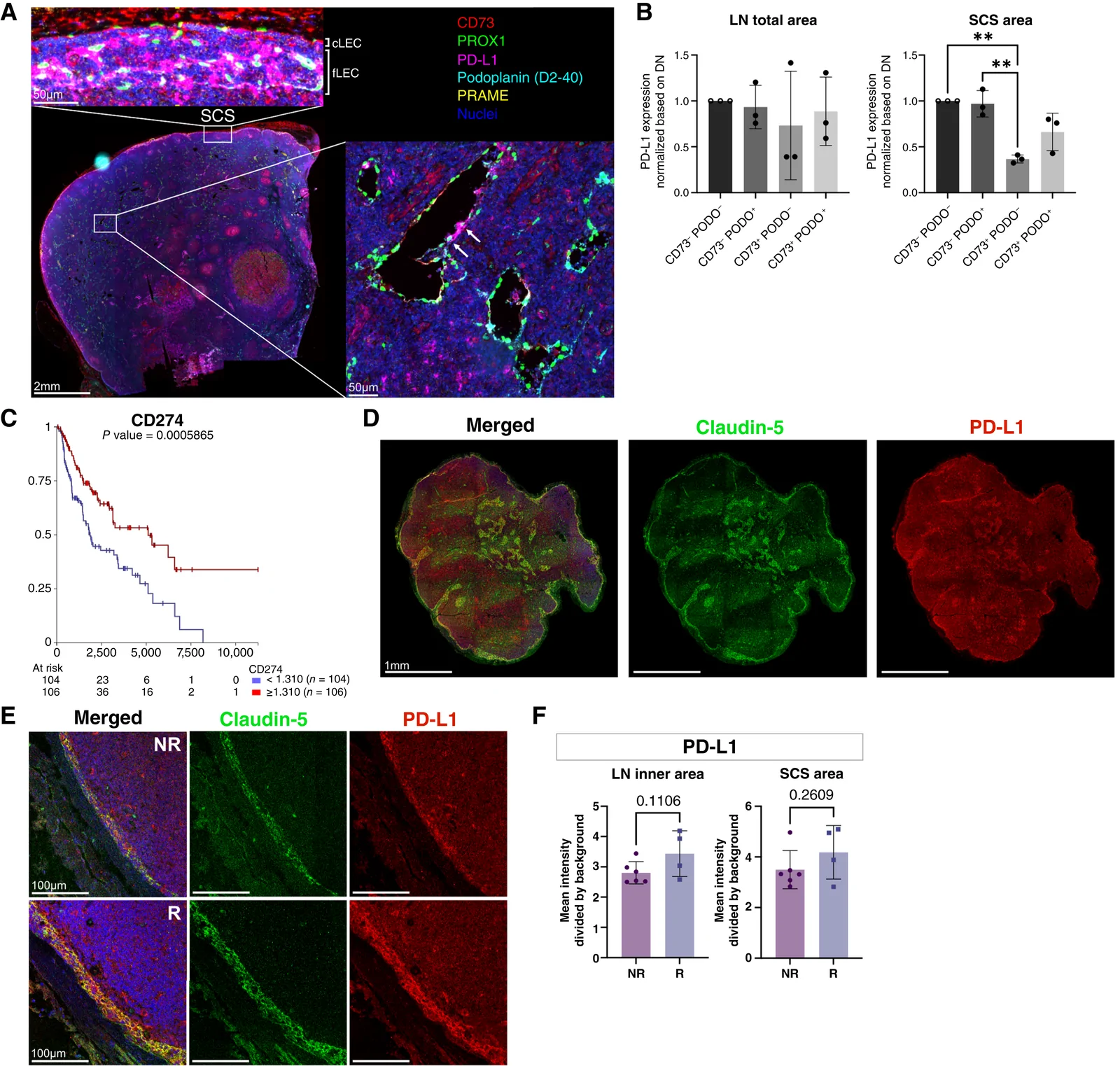
PD-L1 expression by LN LECs in human melanoma–draining LNs. **A,** Representative microscopy images of immunofluorescence staining for CD73 (red), PROX1 (green), PD-L1 (pink), podoplanin (D2-40, light blue), PRAME (yellow), and nuclei (blue) in a human melanoma–draining LN. Zoomed-in views show a representative area of the SCS and the cortical region. **B,** Quantification of PD-L1 mean fluorescence intensity in the total LN area and SCS area by CD73- and podoplanin-defined subsets of PROX1+ LECs (>2,000/patient) after intensity normalization to the double-negative (DN) subset. *N* = 3 patients. **, *P* < 0.01; one-way ANOVA with a Šídák post test. PODO, podoplanin. **C,** Kaplan–Meier curves of *CD274* gene expression correlating with overall survival in human melanoma data (SKCM-The Cancer Genome Atlas dataset, filtered for LN as site of resection/biopsy). The median value of gene expression was used as cutoff. Statistical analysis was conducted using the log-rank test. **D,** Immunofluorescence images of a representative tdLN from a patient with melanoma stained for claudin-5 and PD-L1. **E,** Zoomed-in views from an SCS area of melanoma-draining LNs for representative NR and R patients. **F,** Quantification of PD-L1 mean fluorescence intensity in SCS area and inner LN area by claudin-5^+^ vascular structures after normalization to a low PD-L1 level background. *N* = 4–6 patients/group. Student *t* test.

In murine LNs, constitutive PD-L1 expression by fLECs and mLECs has been demonstrated both at the mRNA and protein levels under steady-state and pathologic conditions. In line with this, single-cell RNA sequencing of metastasis-free LNs from patients with cancer has confirmed PD-L1 transcripts in mLEC and fLECs (27, 28). However, lymphatic PD-L1 expression on the protein level in human LNs has not been demonstrated so far. Therefore, we established a multiplex immunofluorescence staining panel to detect lymphatic PD-L1 expression in tdLNs of patients with melanoma (Fig. 1A). We included PROX1 as a pan-LEC marker, CD73 (NT5E) as a cLEC marker, and podoplanin (D2-40), broadly expressed by peripheral LECs (27). In addition, we stained for PRAME, a melanoma marker, to detect metastasis. PROX1^+^ nuclei were clearly distinguishable in LNs obtained from a small cohort of patients with melanoma (*N* = 3, Supplementary Table S2; Fig. 1A). We noticed that many LECs expressed neither CD73 nor podoplanin, whereas some LECs were single or double-positive. Of note, CD73 faithfully stained cLECs in the SCS region but was not a reliable cLEC marker across the whole LN. Heterogeneous expression could be observed within individual lymphatic sinuses, in which some LECs expressed podoplanin whereas others were CD73^+^, even deep in the LN cortex (Fig. 1A). Mirroring the expression pattern of CD73 and podoplanin, PD-L1 expression exhibited considerable heterogeneity among LECs, showing individual PD-L1^+^ and PD-L1^–^ LECs sometimes even within the same sinus cross-section (indicated by white arrows, Fig. 1A).

For quantification, individual LEC nuclei were selected based on PROX1 staining in either the whole LN cross-section or in the SCS region only (Supplementary Fig. S1A and S1B), and an expanded area around these nuclei was considered as proxy for the LEC cell body. We then classified LECs into four phenotypic subsets based on the expression of CD73 and podoplanin and subsequently determined the mean fluorescence intensity of PD-L1 within each of these subsets. No significant difference in normalized PD-L1 staining intensity could be detected among the four LEC subsets when the entire LN area was taken into account, although two of three LNs showed lower PD-L1 expression in CD73^+^ podoplanin– LECs (Fig. 1B; Supplementary Fig. S1A). In contrast, within the SCS region, PD-L1 expression was consistently observed in fLECs that were double-negative for CD73 and podoplanin, whereas CD73^+^ podoplanin– cLECs showed comparably low PD-L1 expression. Intensity-based quantification across all three patients confirmed that SCS cLECs (CD73^+^ podoplanin^–^) exhibited approximately three-fold lower PD-L1 staining intensity compared with the CD73^−^ subsets (Fig. 1B; Supplementary Fig. S1B). In conclusion, these data demonstrate that PD-L1 is expressed by LECs in human melanoma-draining LNs at the protein level, with CD73^+^ SCS cLECs exhibiting low to no PD-L1 in contrast to fLECs and other LECs in the LN cortex, mirroring the expression pattern of PD-L1 in murine LN LECs.

The PD-L1/PD-1 pathway is an established target for ICB therapy in melanoma, but only a subset of patients shows durable responses toward ICB. To investigate a potential relationship between lymphatic PD-L1 expression and ICB outcomes, we first extracted LN PD-L1 (encoded by the *CD274* gene) expression versus survival of patients with cutaneous melanoma from The Cancer Genome Atlas database and found a strong correlation between high PD-L1 expression and good outcomes (Fig. 1C). As bulk RNA sequencing cannot distinguish PD-L1 expression in individual cell types including LECs, we next stained tdLN sections from a small cohort of patients with melanoma treated at our center who responded or did not respond to adjuvant nivolumab (anti–PD-1) treatment (Supplementary Table S2). PD-L1 expression was quantified within vascular structures identified by the pan-endothelial marker claudin-5 (Fig. 1D; Supplementary Fig. S1C; ref. 29). Interestingly, when we analyzed the SCS region, in which claudin-5^+^ vascular structures predominantly correspond to lymphatic sinuses, PD-L1 staining intensity within these claudin-5^+^ vessels tended to be approximately 20% higher in ICB-R patients compared with NR (not significant, Fig. 1E and F). When we analyzed the LN cortex and inner LN area, a similar, nonsignificant trend toward increased PD-L1 expression in ICB-Rs was observed (Fig. 1F). If substantiated in a larger patient cohort, these results could suggest that lymphatic PD-L1 expression correlates with ICB responsiveness in patients with melanoma. However, our analysis of the inner LN and cortical regions of the LNs likely included blood vessel endothelium as well, and the size of our patient cohort was too small to determine statistical significance.

### Lymphatic PD-L1 deletion does not influence melanoma growth or progression in mice

**Figure 2.**
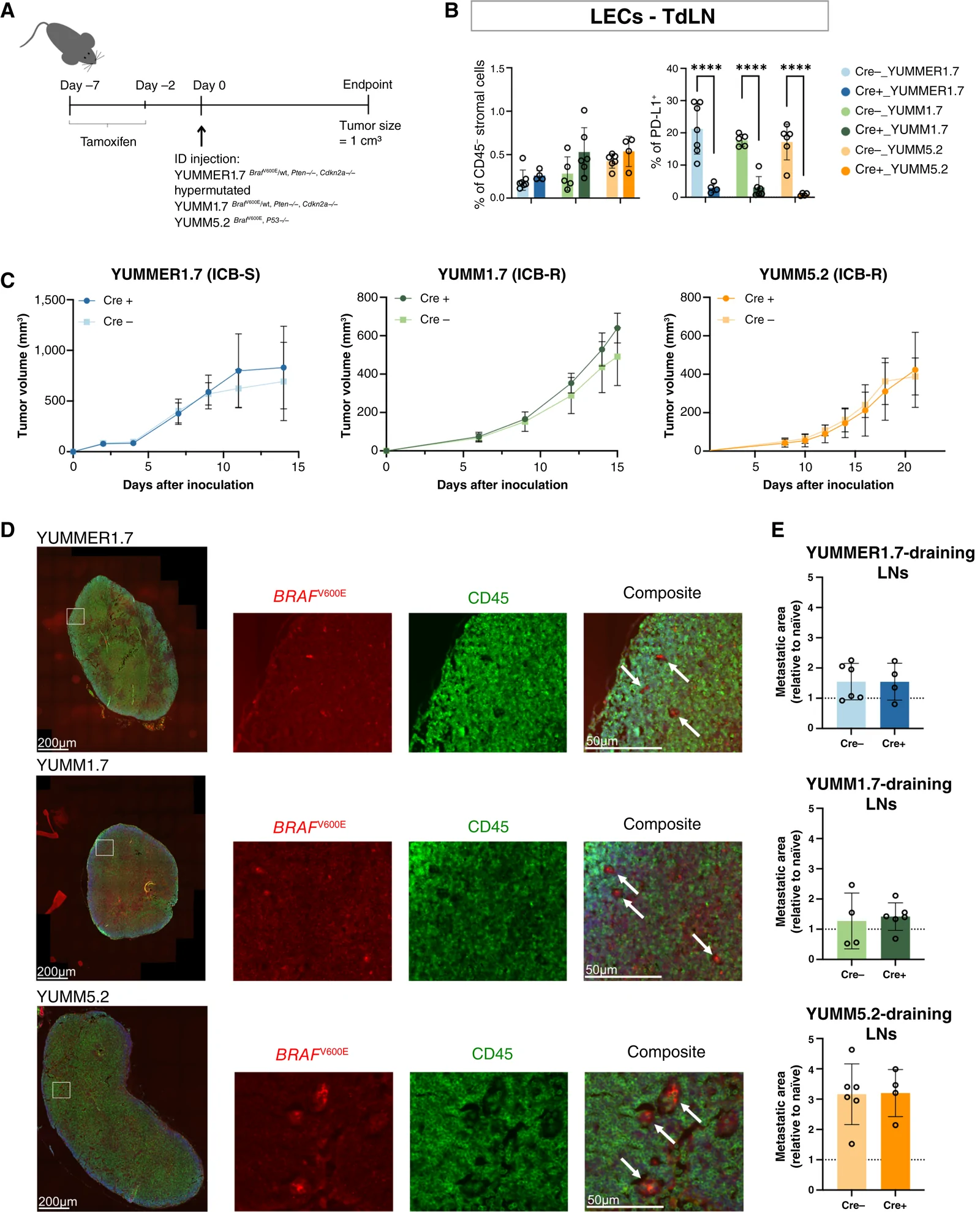
Lymphatic PD-L1 does not affect melanoma tumor growth or metastasis in tdLNs. **A,** Study design overview. PD-L1^LECKO^ mice were treated with tamoxifen for five consecutive days before being inoculated intradermally (ID) with syngeneic melanoma cells (indicated by the arrow). Mice were sacrificed when the tumor volume reached 1 cm^3^. **B,** Frequency of LECs (CD45^−^ podoplanin^+^ CD31^+^) and PD-L1+ LECs in the YUMMER1.7-, YUMM1.7- and YUMM5.2-draining LNs of PD-L1^LECKO^ mice treated with tamoxifen. ****, *P* < 0.0001; two-way ANOVA with a Šídák post test. **C,** Tumor growth curve of YUMMER1.7, YUMM1.7, and YUMM5.2 in PD-L1^LECKO^ mice treated with tamoxifen. Tumor growth was monitored by caliper measurements. *N* = 4–7 mice/group. **D,** Representative microscopy images of immunofluorescence staining for BRAF^V600E^ (red), CD45 (green), and nuclei (blue) of LNs draining the indicated melanoma mouse models. Only the Cre- group is shown. Arrows indicate metastatic nodules. **E,** Quantification of metastatic areas in naïve and melanoma-draining LNs. *N* = 4–7 LNs/group. Dashed line represents background staining obtained using naïve LNs. Student *t* test.

Having confirmed that LN LECs express PD-L1 in patients with melanoma, we next examined the role of LEC-expressed PD-L1 in tumor immunity using clinically relevant mouse models of melanoma. To this end, we selected three syngeneic melanoma models carrying mutations commonly found in human melanoma: YUMM5.2 (*Braf*^V600E/wt^, *P53*^−/−^), YUMM1.7 (*Braf*^V600E/wt^, *Pten*^*−*/−^, *Cdkn2a*^−/−^), and YUMMER1.7 (22, 23), a UV-induced somatically hypermutated derivate of YUMM1.7 that has been described as ICB sensitive because of increased mutational burden, whereas YUMM1.7 and YUMM5.2 are ICB-resistant (22, 30). Consequently, this panel of melanoma models allowed us to assess the impact of lymphatic PD-L1 across distinct ICB-responsiveness settings, mimicking clinical diversity with regard to ICB outcomes in melanoma. Tumor cells were injected intradermally in both flanks of tamoxifen-treated PD-L1^fl/fl^ x Prox1-CreER^T2^ mice (PD-L1^LECKO^ mice), and tumor growth was monitored until the first mouse reached a tumor volume of 1 cm^3^ (Fig. 2A). Flow cytometry confirmed effective PD-L1 deletion on LN LECs at the study endpoint, without affecting their frequency (Fig. 2B; Supplementary Fig. S2A and S2B). Notably, similar to our previous results using the B16-F10 melanoma model (19), deletion of LN LEC-expressed PD-L1 did not affect primary tumor growth in any of the melanoma models tested (Fig. 2C). Next, we investigated the occurrence of metastasis in the respective tdLNs of each melanoma model using an antibody against the *BRAF*^V600E^ mutation of the YUMM cell lines, in combination with CD45 (Fig. 2D and E). Among the models, YUMM5.2 exhibited the highest metastatic burden, whereas YUMM1.7 and YUMMER1.7 cell lines showed similar, lower levels. Deletion of LEC-expressed PD-L1 did not alter the occurrence of tdLN metastasis in any of the models (Fig. 2E).

### Lymphatic PD-L1 deletion increases proliferating PD-1^+^ T cells in ICB-sensitive melanoma

**Figure 3.**
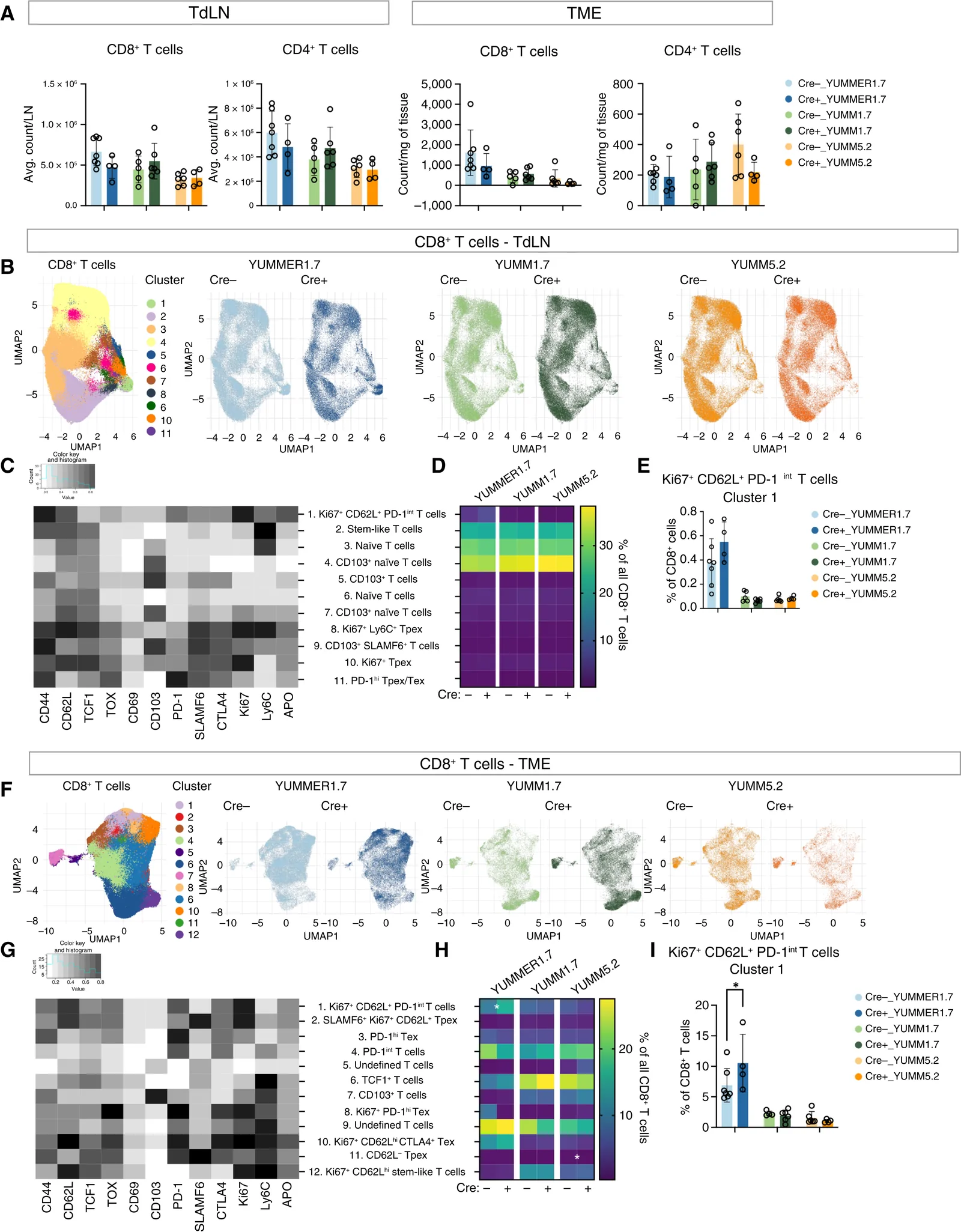
Lymphatic PD-L1 deletion increases proliferating PD-1^+^ CD8^+^ T cells in exclusively ICB-sensitive melanoma. **A,** Average (Avg.) count of CD8^+^ and CD4^+^ T cells per LN in tdLNs and count per mg of primary tumor tissue for the YUMMER1.7, YUMM1.7, and YUMM5.2 melanoma models. *N* = 4–7 mice/group. Two-way ANOVA with a Šídák post test. **B,** UMAP with unsupervised clustering of CD8^+^ T cells in the tdLNs and individual UMAP plots for each model and condition. **C,** Heatmap depicting mean marker expression across all clusters. **D,** Heatmap of the cluster frequencies of CD8^+^ T cells in the tdLNs under the indicated treatment. **E,** Frequency of Ki67^+^ CD62L^+^ CD44^+^ PD-1^+^ CD8^+^ T cells (cluster 1) in tdLNs under the indicated conditions determined by classic flow cytometry gating. *N* = 4–7 mice/group. Two-way ANOVA with a Šídák post test. **F,** UMAP with unsupervised clustering of CD8^+^ T cells in the TME and individual UMAP for each model and condition. **G,** Heatmap depicting mean marker expression across all clusters. **H,** Heatmap of the cluster frequencies of CD8^+^ T cells in the TME under the indicated treatment. *, *P* < 0.05; Student *t* test. **I,** Frequency of Ly6C^–^ Ki67^+^ CD62L^+^ PD-1^+^ CD8^+^ T cells (cluster 1) in the TME under the indicated conditions determined by classic flow cytometry gating. *N* = 4–7 mice/group. *, *P* < 0.05; two-way ANOVA with a Šídák post test. APO, apotracker; T_ex_, exhausted T cell; T_pex_, progenitor exhausted T cell.

To characterize the immune landscape in tdLNs and primary tumors following targeted lymphatic deletion of PD-L1, we collected these tissues at the study endpoint and performed in-depth flow cytometry analysis. Initially, we manually gated CD8^+^ and CD4^+^ T cell populations in tdLNs and primary tumors (Supplementary Fig. S3A). Although YUMMER1.7 tumors showed the highest number of tumor-infiltrating CD8^+^ T cells compared with the other models, no significant changes were observed in neither CD8^+^ nor CD4^+^ T cell counts in tdLNs and in the TME upon lymphatic PD-L1 deletion (Fig. 3A; Supplementary Fig. S3B). Likewise, no significant differences in the relative frequencies of Foxp3^+^ regulatory T (Treg) and helper T cells (Th) could be observed (Supplementary Fig. S3C). To deeply dissect the T cell composition in tdLNs and primary tumors after lymphatic PD-L1 deletion, we applied high-dimensional flow cytometry, including markers of T cell subsets, activation and exhaustion states, followed by unsupervised clustering. In tdLNs, we identified 11 clusters of CD8^+^ T cells (Fig. 3B and C). Across all melanoma models, the dominant clusters of CD8^+^ T cells were a CD44^+^ TCF1^+^ stem-like T cell cluster (cluster 2) and two naïve CD8^+^ T cell clusters (cluster 3 and 4; Fig. 3C and D). Notably, in contrast to YUMM1.7 and YUMM5.2-draining LNs, YUMMER1.7-draining LNs harbored a four to five times elevated proportion of proliferating CD62L^+^ PD-1^int^ T cells (cluster 1), which tended to increase further upon lymphatic PD-L1 deletion (Fig. 3D and E). Within the TME, we identified 12 infiltrating CD8^+^ T cell clusters (Fig. 3F and G). In primary YUMMER1.7 tumors, the frequency of a matching population of proliferating CD62L^+^ PD-1^int^ T cells (cluster 1) was again higher compared with the other models (about 2–3 times) and significantly increased further following lymphatic PD-L1 deletion (Fig. 3H and I). Additionally, a PD-1^hi^ TOX^hi^ cluster of exhausted T cells (cluster 10) was considerably increased in the TME of YUMMER1.7 tumors compared with the other models but was not significantly affected by lymphatic PD-L1 deletion (Fig. 3H). Together, these findings suggest that lymphatic PD-L1 deletion in the YUMMER1.7 model might promote proliferation and/or exit of proliferating CD8^+^ T cells in tdLNs, facilitating their trafficking into the TME in which they differentiate into exhausted T cells. Alternatively, tumor-associated LECs expressing PD-L1 might reduce T cell proliferation directly within the TME. Yet, enhanced accumulation of this T cell subset in the TME upon lymphatic PD-L1 depletion was insufficient to reduce primary tumor growth in the YUMMER1.7 model. In contrast, T cells in ICB-resistant melanomas were hardly affected by lymphatic PD-L1 deletion.

### Lymphatic PD-L1 deletion reduces PD-1^hi^ Th1 cells in the TME of ICB-sensitive melanoma

**Figure 4.**
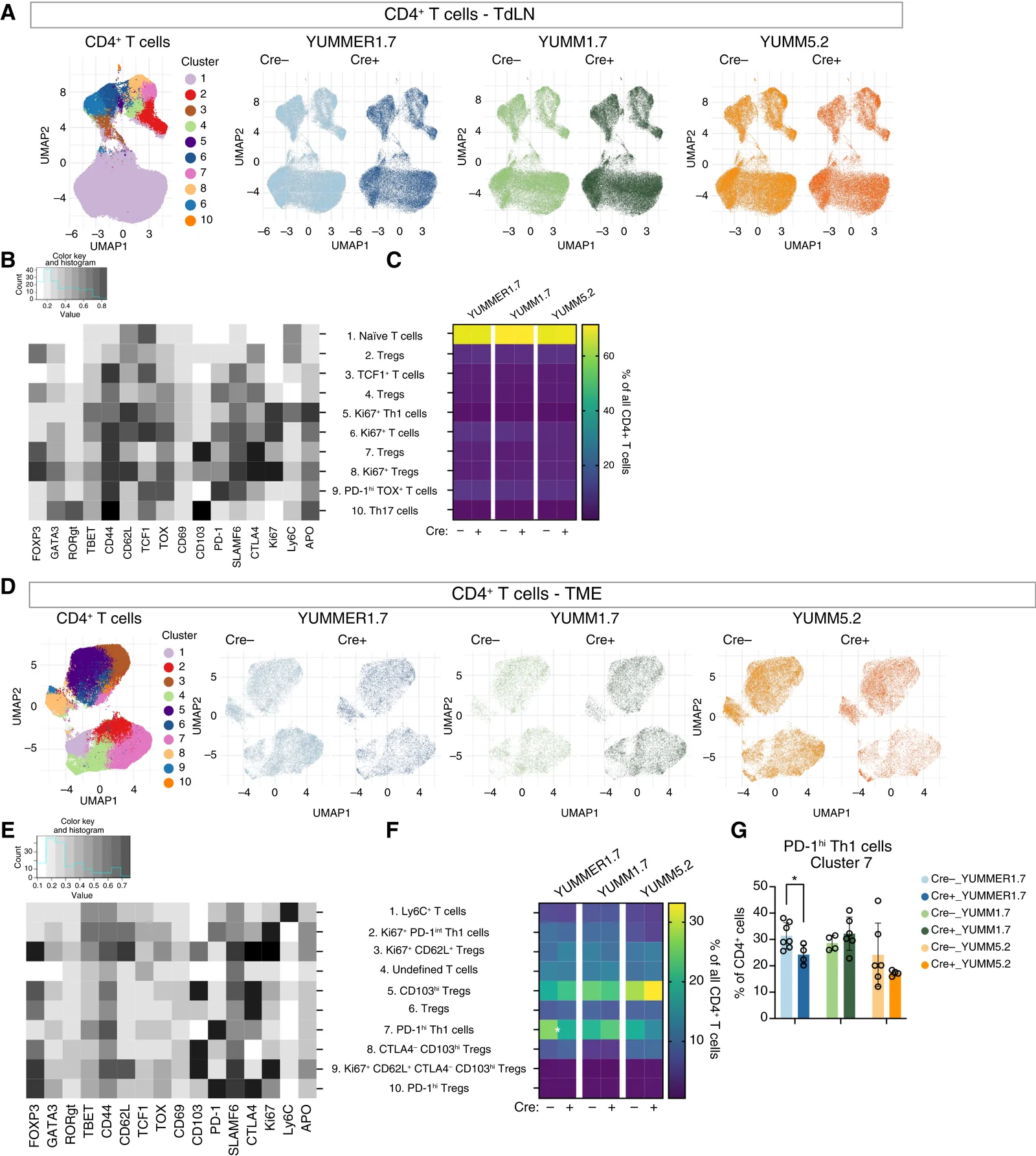
Lymphatic PD-L1 deletion reduces PD-1^+^ Th1 cells in the YUMMER1.7 primary tumor specifically. **A,** UMAP with unsupervised clustering of CD4^+^ T cells in the tdLNs and individual UMAP for each melanoma model and condition. **B,** Heatmap depicting mean marker expression across all clusters. **C,** Heatmap of the cluster frequencies of CD4^+^ T cells in the tdLNs under the indicated treatment. **D,** UMAP with unsupervised clustering of CD4^+^ T cells in the TME and individual UMAP for each melanoma model and condition. **E,** Heatmap depicting mean marker expression across all clusters. **F,** Heatmap of the cluster frequencies of CD4^+^ T cells in the TME under the indicated treatment. *, *P* < 0.05; Student *t* test. **G,** Frequency of PD-1^+^ CD44^+^ Foxp3^^−^^ CD4^+^ T cells (cluster 7) in the TME under the indicated conditions determined by classic flow cytometry gating. *N* = 4–7 mice/group. *, *P* < 0.05; Student *t* test. APO, apotracker.

LECs have been shown to express MHC-I molecules and influence self-reactive and tumor-specific CD8^+^ T cells (9, 19). Besides, LECs also express MHC-II molecules, although it remains unclear whether they can load exogenous peptides themselves or instead acquire peptide–MHC-II complexes from other cells (12, 31, 32). Nonetheless, previous studies have indicated that the MHC-II expression by LECs leads to CD4^+^ T cell dysfunction, by hindering their proliferation and survival, and activation of Tregs (12, 32). Therefore, we investigated whether lymphatic PD-L1 deletion affected CD4^+^ T cell subpopulations in tdLNs and primary tumors. In tdLNs, unsupervised clustering of CD4^+^ T cells resulted in 10 distinct clusters, including a dominant naïve CD4^+^ T cell cluster (cluster 1) that did not show polarization toward any defined CD4^+^ Th subset (Fig. 4A–C). Neither this nor any other cluster, including Tregs, proliferating CD4^+^ T cells, and Th17 cells, was affected by lymphatic PD-L1 deletion (Fig. 4A–C). In contrast, in the TME, unsupervised clustering of infiltrating CD4^+^ T cells resulted in 10 clusters with Foxp3^+^ Tregs (cluster 3, 5, 6, 8, 9, and 10) and a PD-1^hi^ Th1 T cell cluster (cluster 7) representing the dominant CD4^+^ T cell populations (Fig. 4D–F). Interestingly, in YUMMER1.7 tumors, lymphatic PD-L1 deletion specifically led to an approximately 20% reduction of this PD-1^hi^ Th1 T cell cluster (cluster 7; Fig. 4E–G). This finding suggests a potential role of lymphatic PD-L1 in the maintenance of PD-1^hi^ Th1 cells within the TME.

### Lymphatic PD-L1 deletion does not affect the myeloid and B cell populations in tdLNs and the TME

In addition to T cells, LNs and the TME contain abundant B cells and diverse myeloid cell subsets. Although some immune cells are resident within the LNs, many migrate from peripheral tissues to dLNs, in which they play crucial roles in initiating and regulating adaptive immune responses. Recent evidence indicated that PD-1 is expressed by B cell and myeloid cell populations (33–35). This suggests that PD-L1 expressed by LECs could in principle modulate the activities of various immune cell types beyond T cells. Therefore, we analyzed B cell and myeloid cell populations in tdLNs and primary tumors of ICB-sensitive and -resistant melanoma models to determine whether lymphatic PD-L1 deletion affects their composition or phenotype. Upon lymphatic PD-L1 deletion, the frequency and count of myeloid cells in the tdLNs and primary tumor were not affected (Supplementary Fig. S4A and S4B). Similarly, although in-depth flow cytometry analysis using unsupervised clustering of double- or single-positive CD11b^+^ and CD11c^+^ (CD11b^+^/CD11c^+^) myeloid cells from tdLNs and primary tumors indicated that the YUMMER1.7 TME is abundantly infiltrated by granulocytes (cluster 4), no differences following lymphatic PD-L1 depletion were noted (Supplementary Fig. S4C–S4H). Similar to innate myeloid cells, the overall B cell population in tdLNs and the TME was unaffected by lymphatic PD-L1 depletion (Supplementary Fig. S5A and S5B). Unsupervised clustering of the CD19^+^ B cell landscape of tdLNs defined 10 clusters, with a main B cell cluster expressing high levels of activation markers (CD80, CD86) and MHC-II (Supplementary Fig. S5C and S5D). However, none of the B cell clusters were altered upon lymphatic PD-L1 deletion (Supplementary Fig. S5E). Within the TME, the frequency of B cells was too low for a deeper analysis (Supplementary Fig. S5B). Collectively, these results indicate that lymphatic PD-L1 does not influence the composition or phenotypic distribution of myeloid or B cell populations within tdLNs and primary tumors.

### Local regulation of lymphatic PD-L1 on proliferative PD-1^+^ T cells in tdLNs

**Figure 5.**
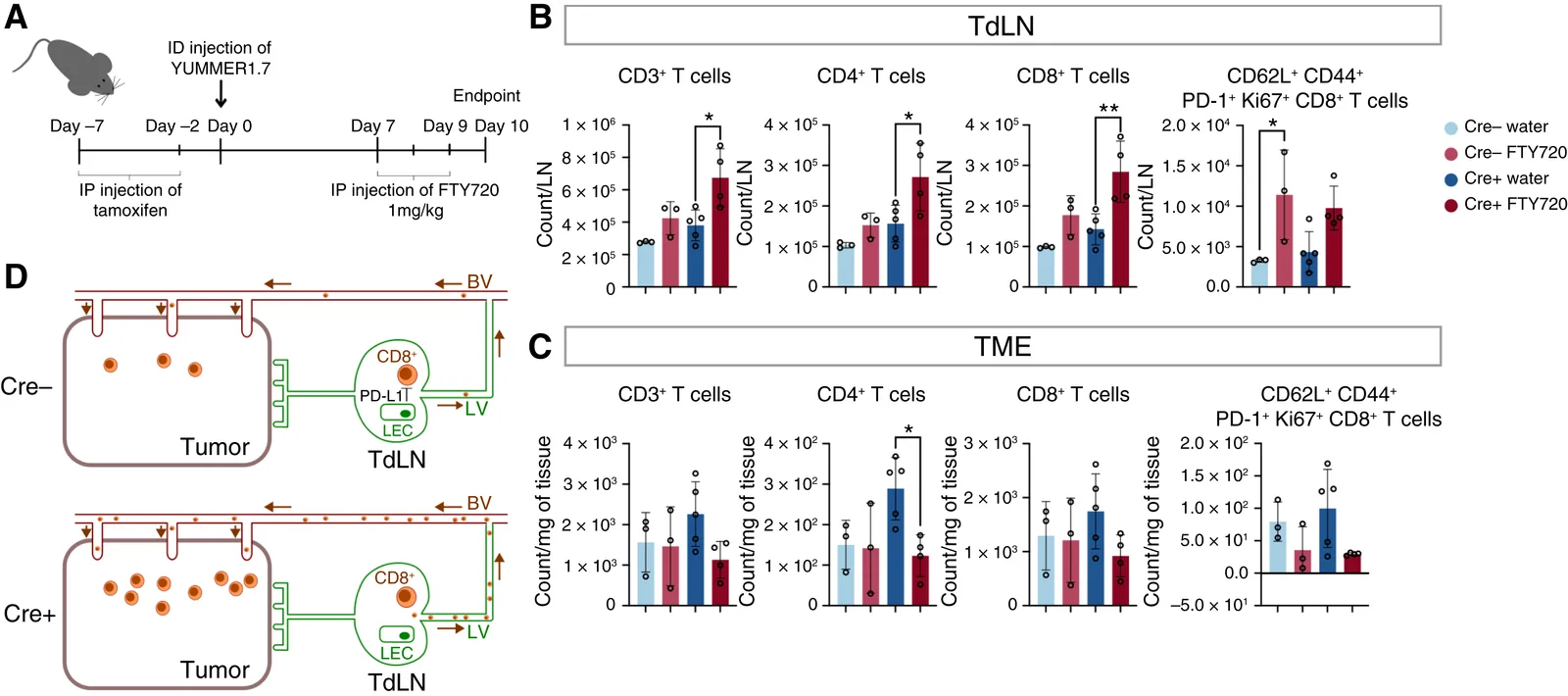
Lymphatic PD-L1 regulates proliferating CD44^+^ CD62L^+^ PD-1^+^ CD8^+^ T cells within YUMMER1.7-draining LNs. **A,** Study design overview. PD-L1^LECKO^ mice were treated with tamoxifen for five consecutive days before being inoculated intradermally with YUMMER1.7 melanoma cells (indicated by the arrow). On day 7, 8, and 9, mice received FTY720 intraperitoneally (IP) before being sacrificed on day 10. **B** and **C,** Count per LN or mg of tumor for CD3^+^ T cells, CD4^+^ T cells, CD8^+^ T cells, and Ki67^+^ CD62L^+^ CD44^+^ PD-1^+^ CD8^+^ T cells in the tdLNs (**B**) and primary tumors (**C**) of the YUMMER1.7 melanoma model under the indicated treatment. *N* = 3–5 mice/group. *, *P* < 0.05; **, *P* < 0.01; one-way ANOVA with a Šídák post test. **D,** Schematic representation of the proposed mode of action. In Cre-mice (top), PD-L1 expressed by LECs in tdLNs inhibits activation, proliferation, or LN egress of early activated CD8^+^ T cells, limiting their migration and accumulation within the TME. In Cre+ mice lacking lymphatic PD-L1 expression, early activated CD8^+^ T cell output of tdLNs is enhanced, resulting in increased infiltration of the TME. BV, blood vessel; LV, lymphatic vessel.

To address whether lymphatic PD-L1 affects CD8^+^ T cell recruitment to the TME by interfering with migration or expansion within tdLNs, or whether PD-L1 in tumor-associated lymphatic vessels suppressed CD8^+^ T cells locally within the TME, we blocked lymphocytes trafficking from tdLNs of YUMMER1.7-bearing PD-L1^LECKO^ mice using FTY720 (Fig. 5A). The tumor growth was not affected by the treatment (Supplementary Fig. S6A). At the endpoint, we harvested tdLNs and tumors to assess the impact of egress blockade on T cell populations within these organs.

As expected, FTY720 treatment increased the number of CD3^+^ T cells in the tdLNs, consistent with effective blockade of lymphocyte egress (Fig. 5B). This increase was driven by the accumulation of both CD4^+^ and CD8^+^ T cells, as the numbers of both subsets were elevated following FTY720 treatment (Fig. 5B). In contrast, the relative frequency of T cells in the tdLNs increased only marginally upon FTY720 treatment (Supplementary Fig. S6B). Curiously, FTY720 treatment of mice lacking lymphatic PD-L1 resulted in even greater, up to two-fold accumulation of CD3^+^, CD4^+^, and CD8^+^ T cells in tdLNs compared with Cre-negative controls (Fig. 5B). In parallel, FTY720 treatment in mice lacking lymphatic PD-L1 reduced the numbers of CD3^+^, CD4^+^, and CD8^+^ T cells in the TME (Fig. 5C).

Next, we examined the impact of FTY720 treatment on the subset of proliferating CD62L^+^ PD-1^+^ CD8^+^ T cells in these organs. Following LN egress blockade, we observed a significant, two- to three-fold accumulation of this specific T cell subset in YUMMER1.7-draining LNs, irrespectively of lymphatic PD-L1 deletion, confirming that these cells proliferate and/or accumulate within the LN and are prevented from exiting (Fig. 5B; Supplementary Fig. S6B). Conversely, within the TME, the number of proliferating CD62L^+^ CD44^+^ PD-1^+^ CD8^+^ T cells decreased upon FTY720 treatment, irrespectively of lymphatic PD-L1 deletion (Fig. 5C; Supplementary Fig. S6C). Thus, the expansion of this specific T cell subset within the TME following lymphatic PD-L1 deletion depends on continuous T cell input from LNs. Collectively, our findings are in line with a model in which lymphatic PD-L1 primarily regulates early steps of tumor-specific T cell activation within tdLNs, with downstream consequences for their accumulation in the TME (Fig. 5D).

## Discussion

In this study, we confirmed that PD-L1 protein is expressed by LECs in human melanoma–draining LNs. We found consistent PD-L1 expression by fLECs and more heterogeneous expression by LECs in the LN cortex, whereas SCS cLECs showed minimal PD-L1 staining, matching the expression pattern in murine LNs. However, our quantification approach has limitations, as we could not faithfully identify the entire cell body of all LEC subsets using the markers included in our staining panel. Although PROX1 staining allowed very specific identification of LEC nuclei, we presumably quantified only a fraction of the PD-L1 staining belonging to each LEC because of their elongated and irregular morphology in tissue sections. Furthermore, robust markers to distinguish LEC subsets in human LNs are lacking. CD73 has been proposed as a cLEC marker (27), but we found that numerous LECs in the LN cortex/medulla were also CD73^+^, complicating the subset assignment of individual LECs. Similarly, podoplanin, which effectively stains LECs in skin and other tissues, showed discontinuous, patchy staining in LNs. To accurately quantify PD-L1 expression across the major LEC subsets in human LN sections, the identification of reliable, subset-specific LEC markers for co-staining will be essential.

Interestingly, LN lymphatic PD-L1 expression correlated with adjuvant ICB responsiveness in a small cohort of patients with melanoma, a finding that may motivate further efforts to determine a potential predictive value of lymphatic PD-L1 expression in the context of ICB therapy. However, evaluation of much larger patient cohorts will be needed to determine whether the level of lymphatic PD-L1 could be suitable as predictive biomarker for ICB in patients with melanoma.

To better understand the functional relevance of lymphatic PD-L1, we deeply and comprehensively characterized the immune landscape of tdLNs and primary tumors in ICB-sensitive (YUMMER1.7) and -resistant (YUMM1.7 and YUMM5.2) melanoma models following genetic deletion of lymphatic PD-L1 in mice. Although the loss of lymphatic PD-L1 did not reduce primary melanoma growth or metastasis, we observed a significant impact on the tumor T cell infiltrate exclusively in the ICB-sensitive YUMMER1.7 model, characterized by an accumulation of proliferating CD62L^+^ PD-1^+^ CD8^+^ T cells and by a reduction of PD-1^hi^ Th1 cells. Early-stage CD8^+^ T cells in tdLNs, particularly stem-like and T_pex_ cells, have been identified as mediators of ICB efficacy. Upon PD-L1/PD-1 blockade, these cells expand and traffic to the tumor, fueling a pool of exhausted T cells within the TME (3, 8, 36, 37). Our results are in line with a model in which lymphatic PD-L1 limits the LN output of such CD8^+^ T cells en route to the TME in “hot”, ICB-sensitive melanoma with high mutational burden and copious tumor-associated antigens available for recognition. Conversely, lymphatic PD-L1 does not exert a significant regulatory role on tumor immunity in ICB-resistant melanoma, perhaps because tumor-associated antigens are scarce and their cross-presentation by LN LECs is limited.

Within tdLNs, we only observed marginal effects of lymphatic PD-L1 deletion on the CD8^+^ T cell landscape, whereas we demonstrated previously that lymphatic PD-L1 promotes expansion of tumor antigen-specific CD44^+^ CD62L^+^ CD8^+^ T cells in tdLNs (19). As the YUMM melanoma models are amelanotic and the immunodominant tumor antigens are unknown, we could only characterize the global CD8^+^ compartment in tdLNs in the present study, missing potential effects of lymphatic PD-L1 on individual tumor antigen-specific CD8^+^ T cell clones. Furthermore, lymphatic PD-L1 might particularly affect T cells transmigrating across cortical lymphatic endothelium and exiting tdLNs via efferent lymphatic vessels, which would result in more pronounced remodeling of the T cell landscapes in the TME compared with tdLNs. In line with this hypothesis, we observed that blocking of lymphocyte egress from tdLNs using FTY720 resulted in exacerbated accumulation of T cells in mice lacking lymphatic PD-L1, compared with controls. Perhaps FTY720-mediated blockade of S1P signaling may lead to retention of T cells in proximity to lymphatic sinuses, extending their exposure to lymphatic PD-L1 which counteracts their activation or proliferation. However, additional experiments to precisely map the localization of individual T cell subsets and PD-L1/PD-1 interactions within tdLNs will be necessary to substantiate this hypothesis.

In addition to CD8^+^ T cells, our data revealed a reduction in a PD-1^hi^ Th1 subset following lymphatic PD-L1 deletion within the primary tumor of the ICB-sensitive YUMMER1.7 model in contrast to the ICB-resistant models. Although the mechanism by which LECs acquire antigen-loaded MHC-II molecules is not entirely clear, previous studies have shown that LECs can impair CD4^+^ T cell proliferation and survival via MHC-II (12, 32). Potentially, IFNγ, a cytokine reported to be abundant in the YUMMER1.7 TME due to high CD8^+^ T cell infiltration (38), may induce MHC-II upregulation on LECs in this model, favoring LEC–CD4 T cell interactions (32). However, the specific mechanism of how lymphatic PD-L1 regulates PD-1^hi^ Th1 cells and the potential impact of this interaction on tumor immunity could not be addressed in our study.

PD-L1 is constitutively expressed at comparably high levels by LN LECs. In contrast, peripheral LECs, such as dermal LECs, typically lack PD-L1 but can upregulate its expression in response to IFNγ stimulation, as observed in tumor-associated lymphatic vessels (17, 18). As tumor-associated lymphatic vessels are generally rare, we could not evaluate whether PD-L1 was efficiently deleted in LECs within the TME in our mouse models. In any case, we cannot exclude that PD-L1 deletion on tumor-associated LECs directly influences the frequency or activation status of T cells within the TME, particularly of those cells recirculating toward tdLNs via the lymphatic system. However, our results obtained after FTY720 treatment suggest that the altered T cell landscape in the TME in response to lymphatic PD-L1 deletion is rather due to differential tdLN T cell output. Taken together, our data show that lymphatic PD-L1 is a significant factor in the regulation of early T cell activation in melanoma-draining LNs and might be relevant for ICB responsiveness in patients with melanoma.

## Supplementary Material

Supplementary Figure S1 Immunofluorescence staining and quantification of PD-L1 in tdLNs of melanoma patients

Supplementary Figure S2 LN LEC analysis by flow cytometry

Supplementary Figure S3 Flow cytometry of T cells in tdLNs and the TME

Supplementary Figure S4 Flow cytometry analysis of myeloid innate immune cells in tdLNs and the TME

Supplementary Figure S5 Flow cytometry analysis of B cells in tdLNs and the TME

Supplementary Figure S6 FTY720 treatment of melanoma-bearing mice

Supplementary Table S1 List of antibodies used for flow cytometry

Supplementary Table S2 Patient characteristics

## Data Availability

The data that support the findings of this study are available in the article, supplementary files, or from the corresponding author upon reasonable request.
