# Supplementary material for: Lymph Node Lymphatic PD-L1 Regulates Tumor-Infiltrating T Cells in Immune Checkpoint–Sensitive Melanoma
Source: Cancer Res Commun. 2026 Oct 23;6(10):2492–504. doi: 10.1158/2767-9764.CRC-26-0666 (PMC13691717; doi:10.1158/2767-9764.CRC-26-0666)
Supplement: Supplementary file 1 [file crc-26-0666_supplementary_figure_s1_suppsf1.pdf]

Supplementary Figure S1.

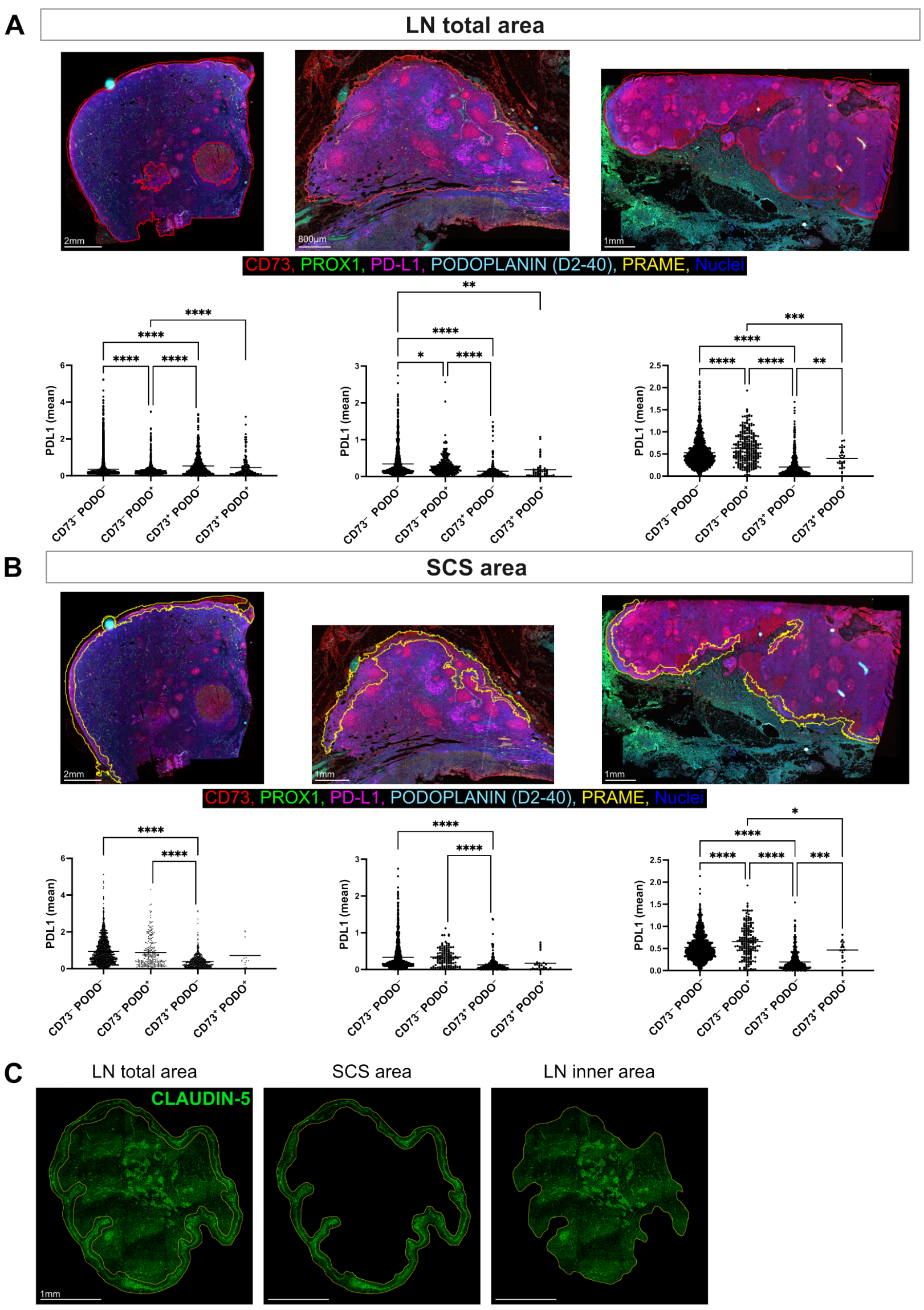

A. Immunofluorescence images of tdLNs from 3 individual melanoma patients stained as in Fig. 1A, with corresponding quantification of PD-L1 mean fluorescence intensity in the total LN area (red lines) by CD73 and PODOPLANIN defined subsets of PROX1$^+$ LECs in each sample separately (bottom left panels), N=2092-14218 cells/group. * P < 0.05; ** P < 0.01, *** P < 0.001; ****, P ≤ 0.0001; one-way ANOVA with Sidak post-test **B.** Images and quantification as in (A), but limited to the SCS area (yellow line), N=676-2265 cells/group. * P < 0.05; *** P < 0.001; ****, P ≤ 0.0001; one-way ANOVA with Sidak post-

test. PODO: PODOPLANIN. **C**. Representative immunofluorescence image for the CLAUDIN-5 channel of a melanoma-dLN from an individual patient as in Fig. 1C. Regions of interest are depicted for the SCS area and the inner LN area (yellow line).
