# Supplementary material for: Lymph Node Lymphatic PD-L1 Regulates Tumor-Infiltrating T Cells in Immune Checkpoint–Sensitive Melanoma
Source: Cancer Res Commun. 2026 Oct 23;6(10):2492–504. doi: 10.1158/2767-9764.CRC-26-0666 (PMC13691717; doi:10.1158/2767-9764.CRC-26-0666)
Supplement: Supplementary file 2 [file crc-26-0666_supplementary_figure_s2_suppsf2.pdf]

Supplementary Figure S2

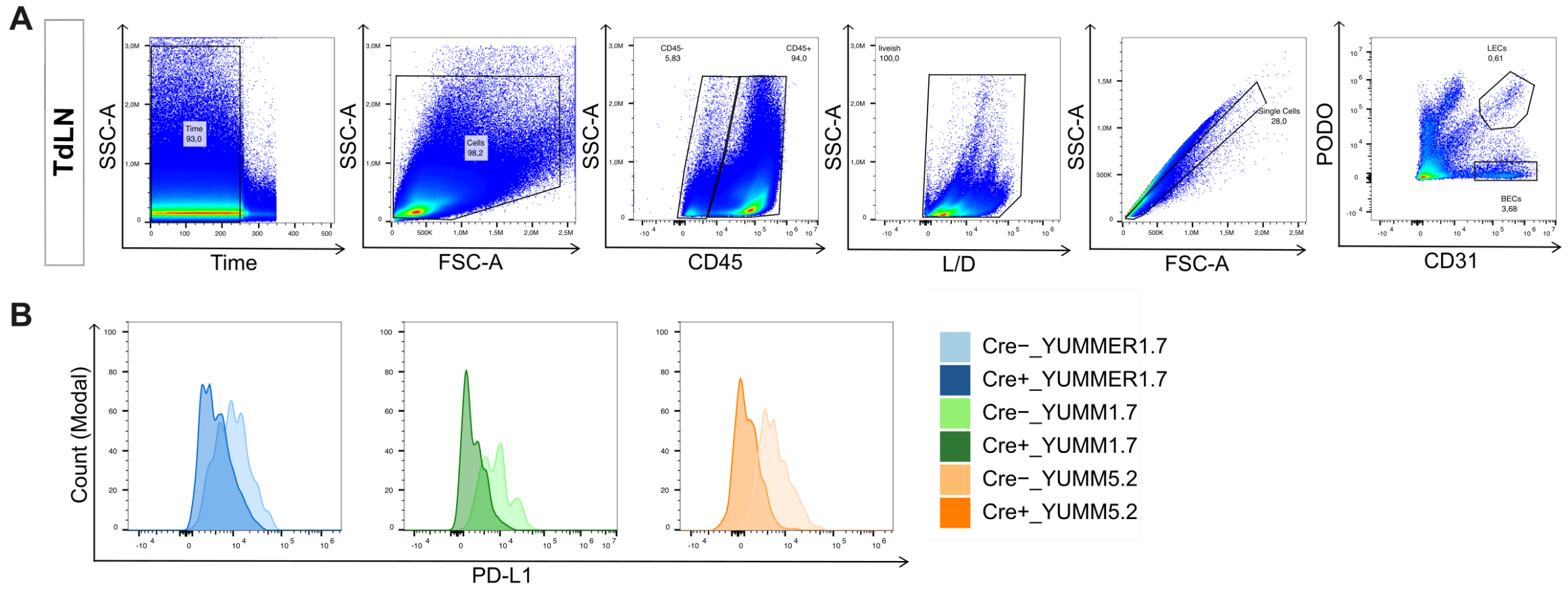

A. Representative gating strategy used to identify LECs illustrated for a YUMMER1.7-dLN.

B. Representative histogram of PD-L1 expression on LN LECs for a Cre- and Cre+ mouse in each of the three melanoma models. TdLN: tumor-draining lymph node. Quantifications are shown in Fig. 2B.
