# Supplementary material for: Lymph Node Lymphatic PD-L1 Regulates Tumor-Infiltrating T Cells in Immune Checkpoint–Sensitive Melanoma
Source: Cancer Res Commun. 2026 Oct 23;6(10):2492–504. doi: 10.1158/2767-9764.CRC-26-0666 (PMC13691717; doi:10.1158/2767-9764.CRC-26-0666)
Supplement: Supplementary file 3 [file crc-26-0666_supplementary_figure_s3_suppsf3.pdf]

Supplementary Figure S3.

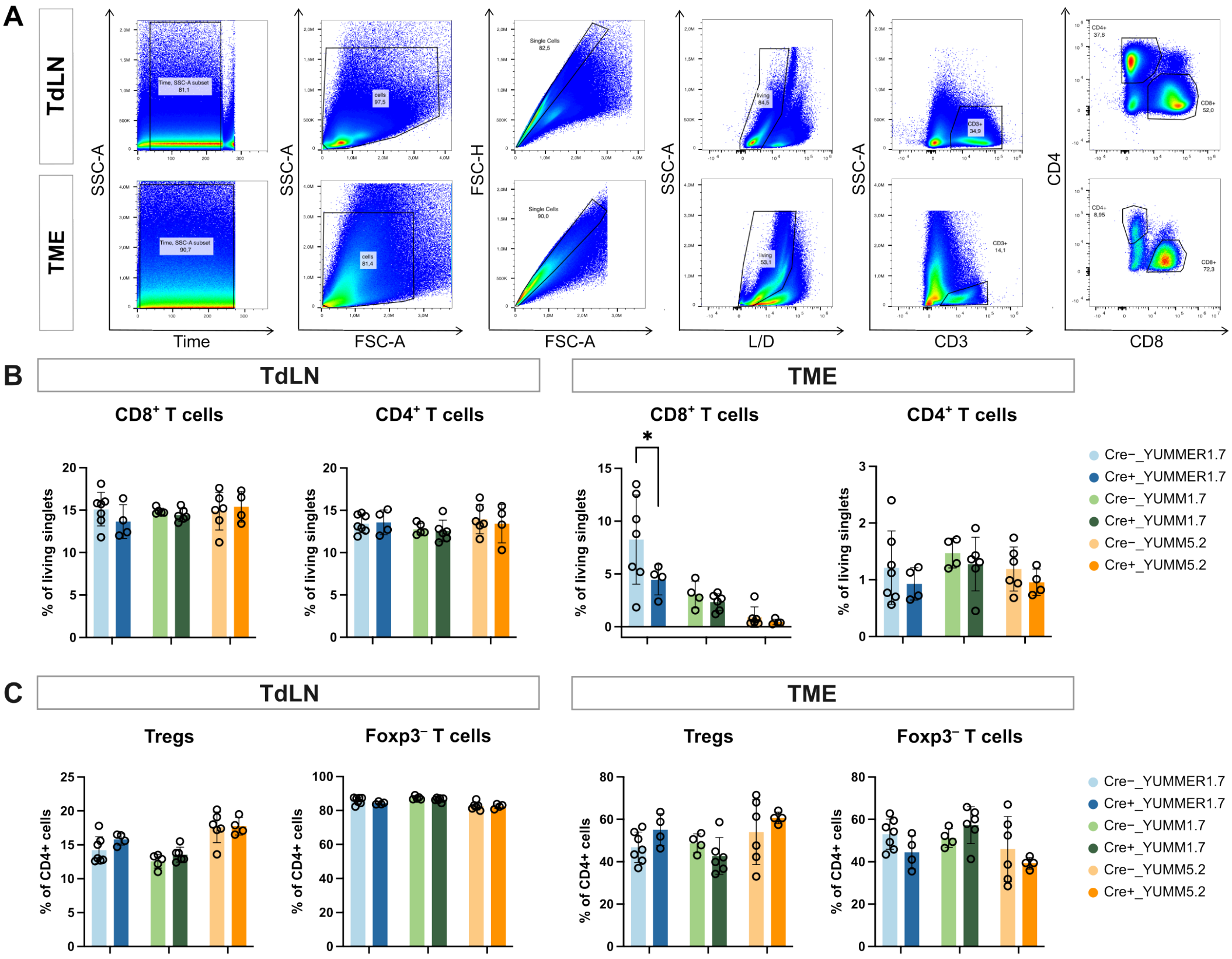

A. Gating strategy used to identify $CD4^+$ and $CD8^+$ T cells illustrated for a YUMMER1.7-dLN and primary tumor. **B**. Frequency of $CD8^+$ and $CD4^+$ T cells in the tdLNs and primary tumors of YUMMER1.7, YUMM1.7 and YUMM5.2 melanoma models. N = 4-7 mice/group. * $P < 0.05$; two-way ANOVA with Sidak post-test. **C.** Frequency of $Foxp3^+$ (Tregs) and $Foxp3^-$ $CD4^+$ T cells in the tdLNs and primary tumors of YUMMER1.7, YUMM1.7 and YUMM5.2 melanoma models. N = 4-7 mice/group. Two-way ANOVA with Sidak post-test. TdLN: tumor-draining lymph node; TME: tumor microenvironment.
