# Supplementary material for: Lymph Node Lymphatic PD-L1 Regulates Tumor-Infiltrating T Cells in Immune Checkpoint–Sensitive Melanoma
Source: Cancer Res Commun. 2026 Oct 23;6(10):2492–504. doi: 10.1158/2767-9764.CRC-26-0666 (PMC13691717; doi:10.1158/2767-9764.CRC-26-0666)
Supplement: Supplementary file 4 [file crc-26-0666_supplementary_figure_s4_suppsf4.pdf]

Supplementary Figure S4.

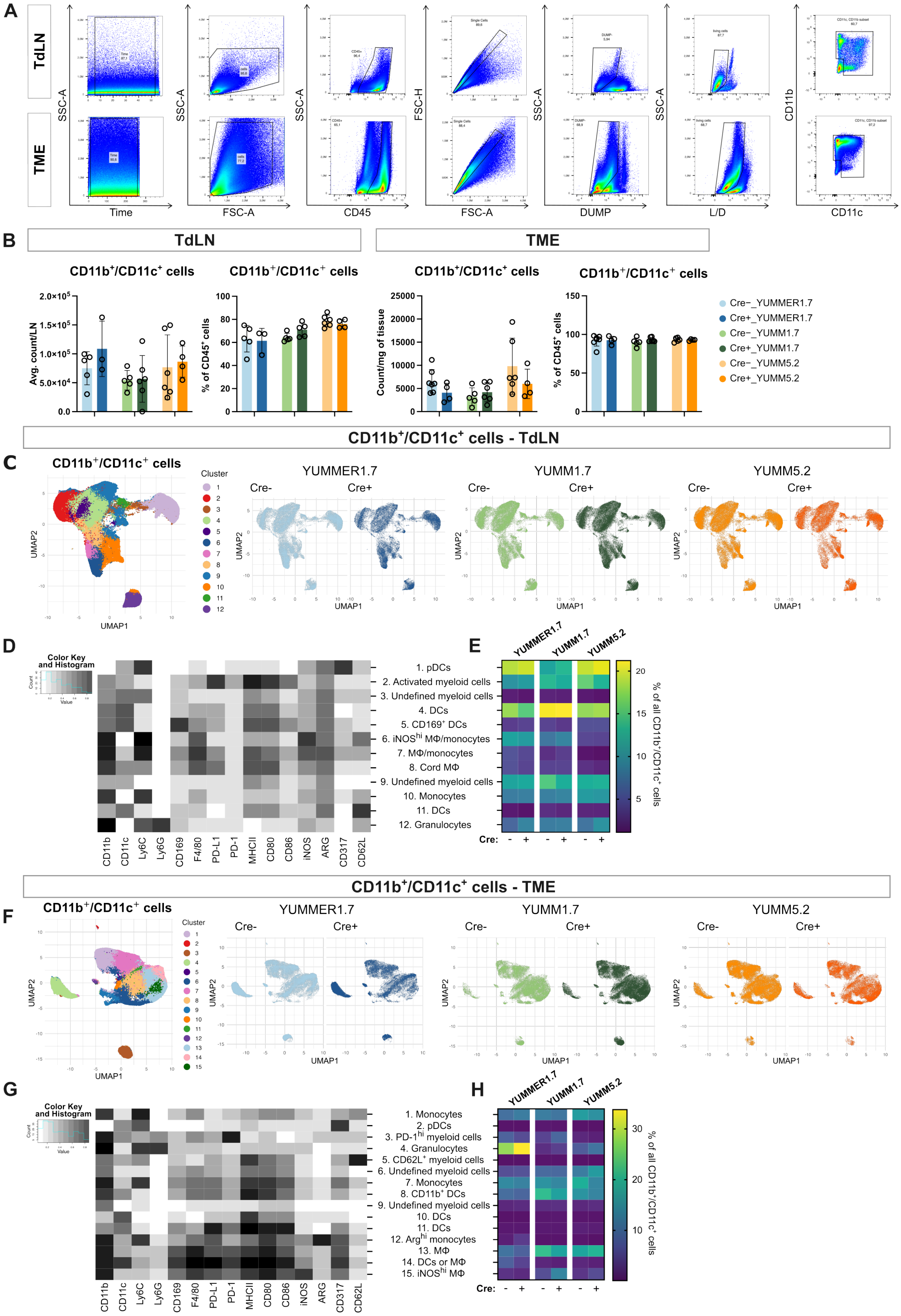

A. Gating strategy used to identify $CD11b^+$ and/or $CD11c^+$ myeloid cells illustrated for a YUMMER1.7-dLN and primary tumor. **B.** Frequency and count (avg. count/LN or count/mg of tumor tissue) of $CD11b^+$ and/or $CD11c^+$ myeloid cells in the tdLNs and primary tumors of YUMMER1.7, YUMM1.7 and YUMM5.2 melanoma models. N = 3-7 mice/group. Two-way ANOVA with Sidak post-test. **C.** UMAP and unsupervised clustering of $CD11b^+$/$CD11c^+$ myeloid cells in the tdLNs and individual cluster plots for each model and condition. **D.** Heatmap representing the mean marker expression across all clusters. **E.** Heatmap of the cluster frequency of $CD11b^+$/$CD11c^+$ myeloid cells in the tdLNs under the indicated treatment. **F.** UMAP and unsupervised clustering of $CD11b^+$/$CD11c^+$ cells in the TME and individual cluster plots for each model and condition. **G.** Heatmap representing the mean marker expression across all clusters. **H.** Heatmap of the cluster frequencies of $CD11b^+$/$CD11c^+$ myeloid cells in the TME under the indicated treatment. TdLN: tumor-draining lymph node; TME: tumor microenvironment; ARG: arginase; MΦ: macrophage; DCs: dendritic cells; pDCs: plasmacytoid dendritic cells.
