# Supplementary material for: Lymph Node Lymphatic PD-L1 Regulates Tumor-Infiltrating T Cells in Immune Checkpoint–Sensitive Melanoma
Source: Cancer Res Commun. 2026 Oct 23;6(10):2492–504. doi: 10.1158/2767-9764.CRC-26-0666 (PMC13691717; doi:10.1158/2767-9764.CRC-26-0666)
Supplement: Supplementary file 5 [file crc-26-0666_supplementary_figure_s5_suppsf5.pdf]

Supplementary Figure S5.

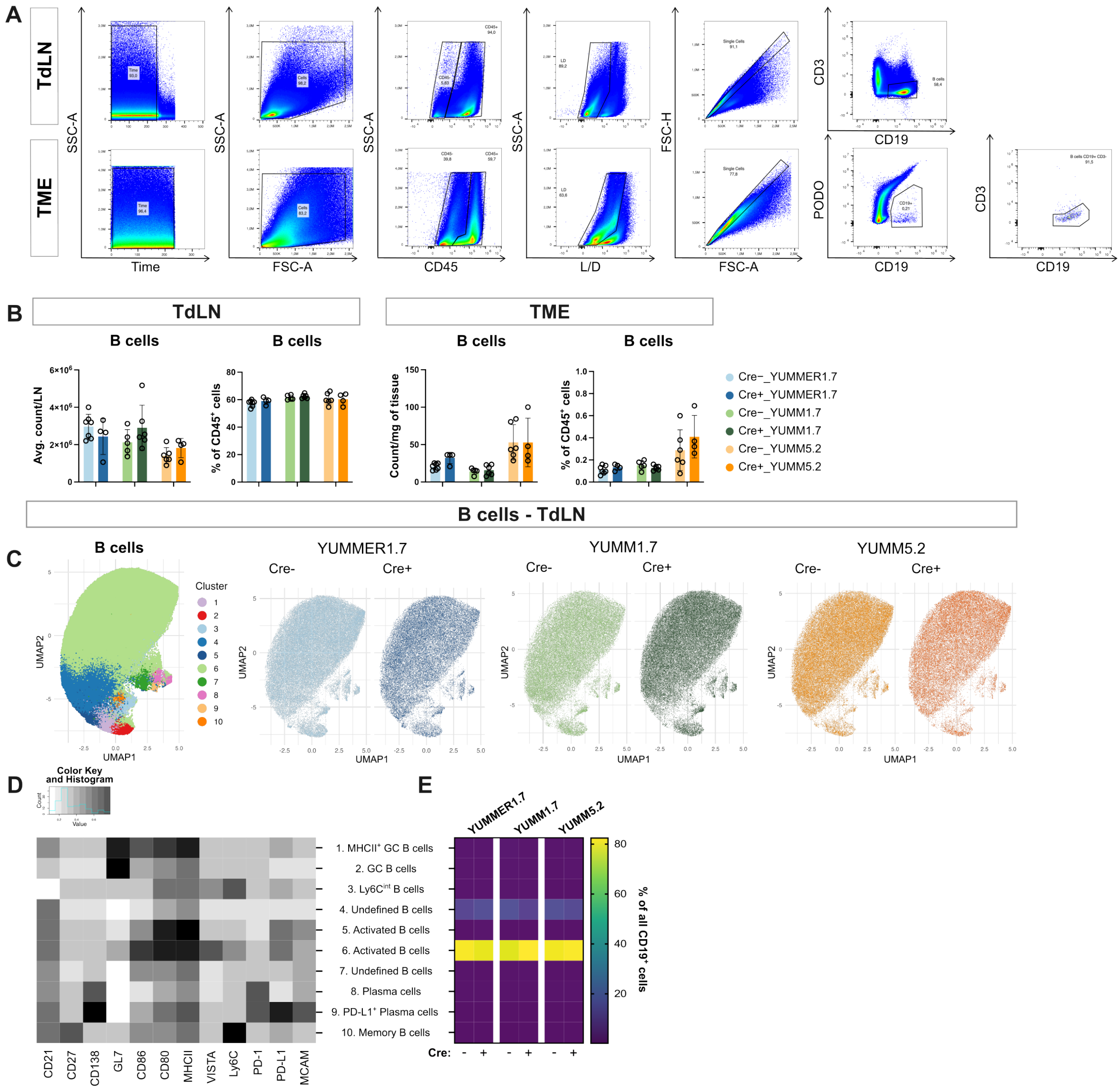

A. Gating strategy used to identify CD19⁺ B cells illustrated for a YUMMER1.7-dLN and primary tumor. **B.** Frequency and count (Avg. count/LN or count/mg of tumor tissue) of CD19⁺ B cells in the tdLNs and primary tumors of each indicated melanoma model. N = 4-7 mice/group. Two-way ANOVA with Sidak post-test. **C.** UMAP and unsupervised clustering of CD19⁺ B cells in the tdLNs and individual cluster plots for each indicated melanoma-dLN and condition. **D.** Heatmap representing the mean marker expression across all clusters. **E.** Heatmap of the cluster frequency of CD19⁺ B cells in the

melanoma-dLNs under the indicated treatment. TdLN: tumor-draining lymph node; TME: tumor microenvironment; GC: germinal center.
