# Supplementary material for: Lymph Node Lymphatic PD-L1 Regulates Tumor-Infiltrating T Cells in Immune Checkpoint–Sensitive Melanoma
Source: Cancer Res Commun. 2026 Oct 23;6(10):2492–504. doi: 10.1158/2767-9764.CRC-26-0666 (PMC13691717; doi:10.1158/2767-9764.CRC-26-0666)
Supplement: Supplementary file 6 [file crc-26-0666_supplementary_figure_s6_suppsf6.pdf]

Supplementary Figure S6.

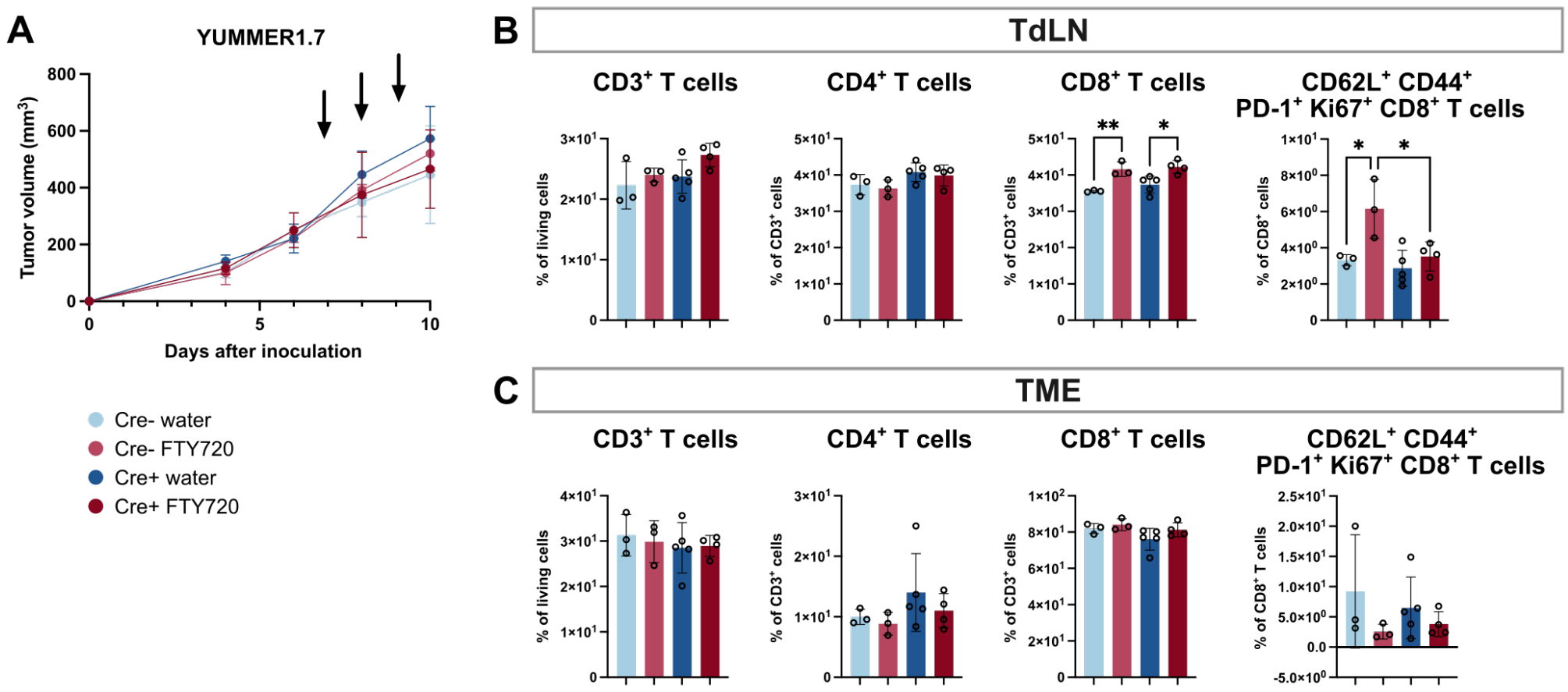

A. Tumor growth curve of YUMMER1.7 melanoma in PD-L1^{LECKO} mice treated with tamoxifen and FTY720 or vehicle control (water). Tumor growth was monitored by caliper measurements. N = 3-5 mice/group. Arrows indicating FTY720 injection. **B-C.** Frequency of CD3$^+$ T cell, CD4$^+$ T cell, CD8$^+$ and Ki67$^+$ CD62L$^+$ CD44$^+$ PD-1$^+$ CD8$^+$ T cell subsets in the tdLNs (**B**) and primary tumors (**C**) of the YUMMER1.7 melanoma model. N = 3-5 mice/group. * P < 0.05; ** P < 0.01; one-way ANOVA with Sidak post-test. TdLN: tumor-draining lymph node; TME: tumor microenvironment.
